# Antibacterial Activity and Mechanisms of Plant Flavonoids against Gram-Negative Bacteria Based on the Antibacterial Statistical Model

**DOI:** 10.3390/ph17030292

**Published:** 2024-02-24

**Authors:** Yu Yan, Xuexue Xia, Aiman Fatima, Li Zhang, Ganjun Yuan, Fengxian Lian, Yu Wang

**Affiliations:** 1 Biotechnological Engineering Center for Pharmaceutical Research and Development, Jiangxi Agricultural University, Nanchang 330045, China; 2 Laboratory of Natural Medicine and Microbiological Drug, College of Bioscience and Bioengineering, Jiangxi Agricultural University, Nanchang 330045, China

**Keywords:** action mode, antimicrobial, *Escherichia coli*, flavonoids, Gram-negative bacteria, Gram-positive bacteria, lipophilicity, LogP, MIC, statistical model

## Abstract

The antimicrobial quantitative structure–activity relationship of plant flavonoids against Gram-positive bacteria was established in our previous works, and the cell membrane was confirmed as a major site of action. To investigate whether plant flavonoids have similar antibacterial effects and mechanisms against both Gram-negative and Gram-positive bacteria, here, the minimum inhibitory concentrations (MICs) of 37 plant flavonoids against *Escherichia coli* were determined using the microdilution broth method, and then the correlation between their lipophilic parameter ACD/LogP or LogD_7.40_ value and their MIC was analyzed. Simultaneously, the correlation between the ACD/LogP or LogD_7.40_ value and the MIC of 46 plant flavonoids reported in the literature against *E. coli* was also analyzed. Both sets of results showed that there is a significant correlation between the LogP value and the MIC of plant flavonoids against Gram-negative bacteria. However, it is difficult to effectively predict the MIC of plant flavonoids against Gram-negative bacteria from their lipophilic parameters. By comparing two regression curves derived from plant flavonoids against Gram-negative and Gram-positive bacteria, it was further discovered that the antibacterial activities of most plant flavonoids against Gram-negative bacteria are stronger than those against Gram-positive bacteria when their LogP values are less than approximately 3.0, but the opposite is true when their LogP values are more than approximately 3.6. Moreover, this comparison also suggests that unlike mainly acting on the cell membrane of Gram-positive bacteria, plant flavonoids have multiple mechanisms against Gram-negative species, while the cell membrane is also an important action site among them. Combined with the correlation analyses between the enzyme inhibitory activity and the LogP value of the reported flavonoids, it was further suggested that DNA gyrase is another important target of plant flavonoids against Gram-negative bacteria.

## 1. Introduction

Antimicrobial resistance (AMR) has been seriously threatening public health and global economic development, and the COVID-19 pandemic has worsened this issue [1]. Therefore, there is an urgent need to discover new antimicrobial agents, especially those against Gram-negative bacteria [2,3,4,5]. Due to the unique structure of the cell envelope, the development of antibacterial agents against Gram-negative bacteria has always been a challenge [3,4]. Fortunately, via the structural modification of tetracyclines, quinolones, aminoglycosides, etc., several antibiotics have been approved for marketing, such as plazomicin and delafloxacin [4,5,6]. Simultaneously, some natural antibacterial products have also been undergoing clinical research [5,6]. Eventually, the resistant mutants will emerge after they spend some time circulating in practice, especially in hospitals [7]. Meanwhile, some plant secondary metabolites have been gradually gaining attention since they not only have antimicrobial activities but also show a good safety profile [8,9]. Among these, plant flavonoids are increasingly gaining widespread recognition [10,11,12,13,14,15].

Flavonoids are an important class of secondary metabolites widely distributed in various plants, and many of them have different degrees of inhibitory activity against numerous pathogenic bacteria. Above all, some of them can enhance the antimicrobial activities of antimicrobial agents and/or even reverse AMR [16,17]. Various antibacterial mechanisms, involving the inhibition of DNA, proteins, and cell envelope biosynthesis, and the damage to the cell membrane, have been reported for plant flavonoids [16,17]. Simultaneously, many structure–activity relationships have been summarized, while some of them contradict one another [17,18,19]. Recently, the antimicrobial quantitative relationship of plant flavonoids against Gram-positive bacteria has been established that [20], through the statistical analyses and validation of large sample sets. Based on this relationship, the minimum inhibitory concentration (MIC) of plant flavonoids against Gram-positive bacteria can be predicted from their lipophilic physicochemical parameter LogP (Log_10_ of partition coefficient) or LogD_7.40_ (Log_10_ of distribution coefficient at pH 7.40), which is calculated from their chemical structures using the software ACD/Labs. Moreover, it has been indicated that the cell membrane is the major action site of plant flavonoids against Gram-positive bacteria, which includes damage to phospholipid bilayers and respiratory chain inhibition [20,21]. Also, experiments interfering with menaquinone-4 or menaquinones extracted from *Staphylococcus aureus* further suggest that the quinone pool is a key target [22].

Considering the difficulties and biological obstacles in discovering natural products with strong antibacterial activity toward Gram-negative bacteria, it was spontaneously wondered from above whether the antimicrobial activities of plant flavonoids against Gram-negative bacteria are also related to their lipophilicities, and whether the antimicrobial quantitative relationship could also be established. In addition, do plant flavonoids have similar antibacterial effects and mechanisms against both Gram-negative and Gram-positive bacteria? The aim to explore these above will enable the prediction of Gram-negative bacteria MIC values and speed up the discovery of novel antimicrobial compounds. Therefore, the correlation between the lipophilic physicochemical parameter (LogP or LogD_7.40_) and the MIC, the antibacterial quantitative structure–activity relationship, and the possible mechanisms of plant flavonoids against Gram-negative bacteria were further explored by following a procedure similar to those used to explore Gram-positive species.

## 2. Results

### 2.1. Structure, Antibacterial Activity, and Physicochemical Parameters

**Figure 1:**
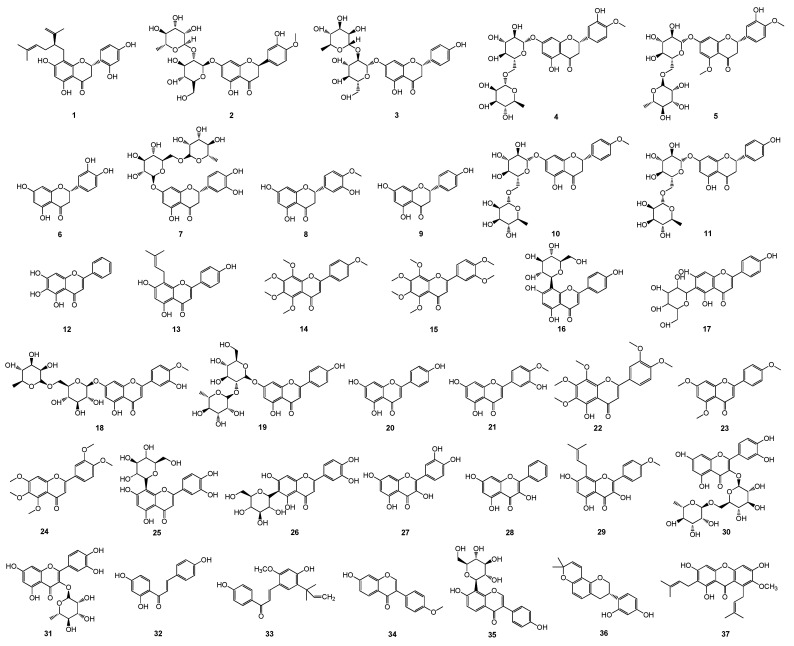
Chemical structures of 37 tested plant flavonoids.

A total of 37 plant flavonoids (Table 1 and Figure 1), with seven structural subtypes, namely, dihydroflavones, flavones, flavonols, chalcones, isoflavones, isoflavanes, and xanthones, were selected for the antimicrobial susceptibility assay and correlation analyses of the antimicrobial activity MIC and the lipophilic physicochemical parameter LogP or LogD_7.40_. Their MIC values (μM) against Escherichia coli and *S. aureus* were determined, and they are listed in Table 1, along with their calculated LogP and LogD_7.40_ values. The results show that all these flavonoids had very weak antimicrobial activities against *E. coli*, with the MIC ranging from 1206.15 to more than 6820.53 μM. This indicates that these plant flavonoids, with LogP and LogD_7.40_ values ranging from 1.26 to 6.70, have weak inhibitory activity against Gram-negative bacteria. However, they presented various degrees of antibacterial activities against *S. aureus*, with the MIC ranging from 9.42 to 13,552.14 or more than 7578.45 μM. According to the previously reported antimicrobial quantitative relationship, the MIC values of these plant flavonoids against Gram-positive bacteria *S. aureus* were simultaneously calculated, and the results show that 81.1% of the calculated MIC values fell into the acceptable range of 1/8 to 8 of the test MIC ones (Table 1). This further confirms that the antimicrobial activity of plant flavonoids against Gram-positive bacteria can be effectively calculated or predicted. 

Also, the MIC values of 52 plant flavonoids (Figure S1) against Gram-negative bacteria *E. coli* were collected from 16 papers in order to use additional data to obtain more reliable conclusions, and they are presented in Table 2. These plant flavonoids include eight structural subtypes, such as dihydroflavones, flavones, biflavones, flavonols, chalcones, isoflavones and xanthones. As intuitively observed from Table 2, the flavonoids with LogP values ranging from 1.53 to 8.03 presented various degrees of antibacterial activities against E. coli, with the reported MIC values ranging from 1.49 to 3308.19 or more than 1722.53 μM.

### 2.2. Correlation and Regression Analyses of the Antibacterial Activity and the Physicochemical Parameter

**Figure 2:**
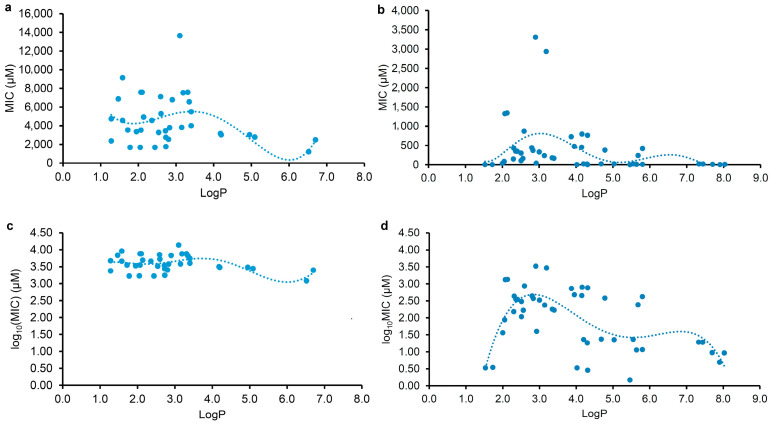
Polynomial regression analyses for the physicochemical parameter LogP (*x*) and the MIC (up) or log_10_(MIC) (down) (*y*) against Gram-negative bacteria, mainly *E. coli*, of plant flavonoids tested ((**a**,**c**), *n* = 37) and reported ((**b**,**d**), *n* = 46).

To examine the correlations between the physicochemical parameter (LogP or LogD_7.40_) and the MIC of plant flavonoids against Gram-negative bacteria, the flavonoids mentioned in Table 1 and Table 2 were selected for statistical analyses. Since the antimicrobial activities of a compound against different pathogenic bacteria vary, even against the same one under different determination conditions, the correlation and regression analyses of the lipophilic parameter LogP or LogD_7.40_ (*x*) and the MIC or log_10_(MIC) (*y*) were individually performed for the tested and reported flavonoids. The regression equations, together with their correlation coefficients (*r*) and coefficients of determination (*R*^2^), are listed in Table 3, and the regression curves from the LogP and the MIC or log_10_(MIC) are shown in Figure 2. According to statistics, the closer the *r* value to 1, the stronger the correlation. Whether there is a significant correlation between two events or not should be tested with *r*-test. An *r* value higher than the critical value of *r*_0.975_(*n* − 2) or *r*_0.995_(*n* − 2) indicates that there is a significant correlation between two events. It could be determined from Table 3 that all the r values for Equations (1)–(6) were higher than the corresponding critical values of *r*_0.975_(35) and *r*_0.975_(44). This indicates that there are significant correlations between the lipophilic parameter LogP or LogD_7.40_ (*x*) and the MIC or log_10_(MIC) (*y*) of plant flavonoids against *E. coli*. However, the LogP presents a better correlation than the LogD_7.40_ with the MIC, as the *r* value of equation (1) was higher than that of Equation (3). This was also confirmed by the fact that there was no significant correlation between the LogD_7.40_ and the MIC of reported plant flavonoids against *E. coli*, as the *r* value of regression Equation (7) was less than the critical one of *r*_0.975_(44). Therefore, the above results, from whether plant flavonoids tested in the current study or those reported in the literature, indicated that the antibacterial activities of plant flavonoids against Gram-negative bacteria are related to their lipophilicities. However, all *R*^2^ values for Equations (1)–(6) were less than 0.45 and far less than 1.0. According to statistics, the closer *R*^2^ to 1, the higher the goodness of fit, and the closer the calculated value, on the whole, to the actual one. Thus, the smaller *R*^2^ values indicate that the goodness of fit of these regression equations is too low to be effectively used to predict the MIC values of a certain plant flavonoid against Gram-negative bacteria.

As shown in Figure 2, both regression curves (Figure 2a,c) from the MIC or log_10_(MIC) and LogP value of the tested plant flavonoids present a biconcave character, and they have two minimum values. However, another two regression curves (Figure 2b,d) from those of reported plant flavonoids have three minimum values. Excluding the difference in the range of the LogP value, it was found that all of them presented similar characters when the LogP values of the plant flavonoids were less than 7.0. Specifically, the left two curves had minimum MIC or log_10_(MIC) values when the LogP values of the tested plant flavonoids were approximately 2.0 and 6.0, whereas the right two curves had minimum MIC or log_10_(MIC) values when the LogP values of the reported plant flavonoids were approximately 1.5 and 5.5. Differently, the right two curves had the third minimum MIC or log_10_(MIC) values. This was mainly attributed to the fact that the LogP values of some of the reported plant flavonoids were more than 7.0. Although there was a slight difference between the curve shapes in Figure 2a,c, another effective fitting equation, Equation (6), with a smaller *r* and *R*^2^ than Equation (5) could also be established from the LogP and log_10_(MIC) of the reported plant flavonoids, and its regression curve (Figure S2) was similar to that in Figure 2c. Therefore, this indicates that maintaining appropriate lipophilicity, probably with a LogP value of approximately 2.0 (or 1.5), 6.0 (or 5.5), or 7.8, is necessary for plant flavonoids to obtain stronger antibacterial activities against Gram-negative bacteria, unlike against Gram-positive bacteria, in which case the higher the LogP value (from 2.0 to 8.9), the stronger the antimicrobial activity presented overall. 

### 2.3. Different Antibacterial Effects and Mechanisms of Plant Flavonoids against Gram-Negative and Gram-Positive Bacteria

Due to various factors, including the method and details of the MIC test, which influence the experimental results, it is usual to observe a significant variation in the MIC values against the same pathogen in different labs. Therefore, the MIC data tested in our lab should be more consistent than the data collected from the literature, and so the regression curve in Figure 2c was further selected for comparison with that from the LogP and log_10_(MIC) of plant flavonoids against Gram-positive bacteria in our previous study [20] to determine their differences. 

**Figure 3:**
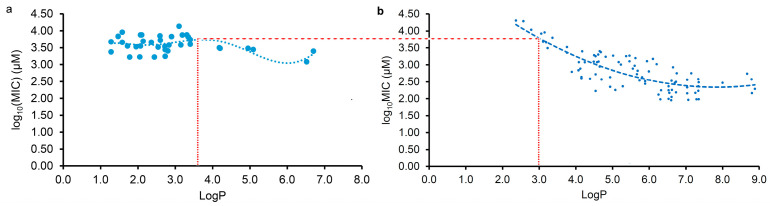
Comparing the regression curves from the LogP and log_10_(MIC) of plant flavonoids against Gram-negative (**a**) and Gram-positive bacteria (**b**). (**a**) same to Figure 2c; (**b**) same to the left figure on Figure 3 of our previous work [20].

As observed in Figure 3, the obvious difference between both curves is that most flavonoids likely have stronger antimicrobial activities against Gram-negative bacteria than Gram-positive species when the LogP value is less than approximately 3.0. However, most flavonoids likely have stronger antimicrobial activities against Gram-positive bacteria than Gram-negative species when the LogP value was more than approximately 3.6. Moreover, the biconcave (Figure 3a) or even triple concave (Figure 2d) character of the regression curve indicates that there are probably multiple action mechanisms of plant flavonoids against Gram-negative bacteria, while the mechanism of plant flavonoids mainly acting on the cell membrane of Gram-positive bacteria only presents a single concave character. As both curves exhibit a similar decreasing trend with the increase in the LogP values ranging from approximately 3.6 to 6.0 (Figure 3), the cell membrane is most likely an important target site for some plant flavonoids against Gram-negative bacteria, especially for those with LogP values ranging from approximately 3.6 to 6.0 (Figure 2c or Figure 3a) or approximately 2.8 to 6.0 (Figure 2d). 

## 3. Discussion

To predict the antimicrobial activities of plant flavonoids against Gram-negative bacteria and to discover compounds with effective inhibitory activity against Gram-negative bacteria, correlation and regression analyses were performed individually using 37 tested and 46 reported data pairs comprising the LogP (or LogD_7.40_) values and MICs of plant flavonoids against Gram-positive bacteria. The results indicate that, similar to those against Gram-positive bacteria, the antibacterial activities of plant flavonoids against Gram-negative species are related to their lipophilicities. However, the antimicrobial quantitative relationship could not be established for plant flavonoids against Gram-negative bacteria, and so their antimicrobial activities against Gram-negative bacteria could not be predicted from their LogP values. Moreover, different from mainly acting on the cell membrane of Gram-positive bacteria, it was further proposed that plant flavonoids have multiple mechanisms against Gram-negative bacteria, while the cell membrane is also an important action site among them.

Lipophilicity, generally expressed as LogP, is a very important descriptor indicating membrane permeation. LogD, which refers to a pH-dependent mixture of all electrical species at any given pH value, is considered a better descriptor reflecting the actual partitioning and lipophilicity. LogD_7.40_ is the LogD at the pH value of 7.40 which is approximately equal to that in human blood or the media of the MIC test. Both lipophilic parameters are important for drug effects. In our previous study [21], various physicochemical parameters of plant flavonoids were investigated to analyze the correlation between them and the MIC of plant flavonoids against Gram-positive bacteria, and the results indicate that there is a good correlation only between the parameter LogP or LogD_7.40_ and the MIC of plant flavonoids against Gram-positive species. Based on this, both physicochemical parameters were selected for the correlation and regression analyses and to conveniently compare the results from plant flavonoids against Gram-negative and Gram-positive bacteria.

As the structural skeleton of plant flavonoids is similar to that of quinolone antimicrobial agents, 14 flavonoids had been designed and synthesized in a previous study [39]. An evaluation of the DNA gyrase inhibitory and antibacterial activities showed that the antibacterial activities of these flavonoids did not parallel the potency at the enzyme level, and the inhibition of DNA gyrase was only one possible reason for this [39]. Therefore, the study concluded that some mechanisms other than DNA gyrase inhibition may play a role in the antibacterial activity of flavonoids. As inferred in our previous study [20,21], the antibacterial activities of plant flavonoids against Gram-positive bacteria mainly depend on their lipophilicities and not on the inhibition of DNA gyrase as the cell membrane is a major site where plant flavonoids act on these bacteria. Inspired by the multiple action mechanisms of plant flavonoids against Gram-negative bacteria, the LogP values of those designed flavonoids were calculated to explore the correlation between their enzyme inhibitory activities and their LogP values, which ranged from 2.07 to 3.76. Surprisingly, the results (Supplementary Tables S2 and S3) demonstrate a significant correlation between the enzyme inhibitory activities and LogP values of the plant flavonoids, and the character of the obtained regression curve (Figure S3) was very similar to that of the curve (Figure 2a or Figure 2c) with LogP values ranging from 2.07 to 3.76. Furthermore, the antibacterial activities of these flavonoids against Gram-negative bacteria, especially *E. coli*_ss_, completely paralleled the potency at the enzyme level. Although no specific MIC values were provided for those flavonoids against some pathogenic strains of Gram-negative bacteria, their relative antibacterial activities could be roughly obtained from the corresponding MIC values in combination with polymyxin B nonapeptide (PMBN) [35]. As a result, it was further suggested that DNA gyrase is another important target of plant flavonoids against Gram-negative bacteria, which is also supported by other studies [28,40,41,42].

According to this, the antibacterial activities of plant flavonoids against Gram-positive bacteria should also present a concave feature when the LogP values are less than approximately 3.0 (Figure 3b). Unfortunately, the LogP value of 3.0 is close to the boundary one, which could possibly cause the curve character to be inconsistent with actual one when the LogP values are less than approximately 3.0 (Figure 3b). This might be similar to the difference in the main target of action of quinolones against Gram-negative (DNA gyrase) and Gram-positive bacteria (Topoisomerase IV). Additionally, their antibacterial activities caused by the inhibition of DNA gyrase were weak overall, according to Figure 2c and Figure 3a. 

Along with the increase in the LogP values, the antibacterial activities of the plant flavonoids against both Gram-negative and Gram-positive bacteria increased when the LogP values were higher than approximately 3.6 (Figure 3a,b). However, their antibacterial activities against Gram-positive bacteria increased more quickly than against Gram-negative species. This might be attributed to the difference in the cell envelope structure and the quinone pool composition in the respiratory chain between Gram-negative and Gram-positive bacteria (explained in the following paragraph) [22]. As mentioned in our previous research [22], menaquinones (MKs) are the sole quinones for electron transfer in the respiratory chain of Gram-positive bacteria, while two types of quinones, namely, MKs and ubiquinones, have been discovered in that of Gram-negative bacteria. This may be an important reason why the plant flavonoids showed weaker antimicrobial activities against Gram-negative than against Gram-positive bacteria when the LogP values were more than approximately 3.6 (Figure 3a).

**Figure 4:**
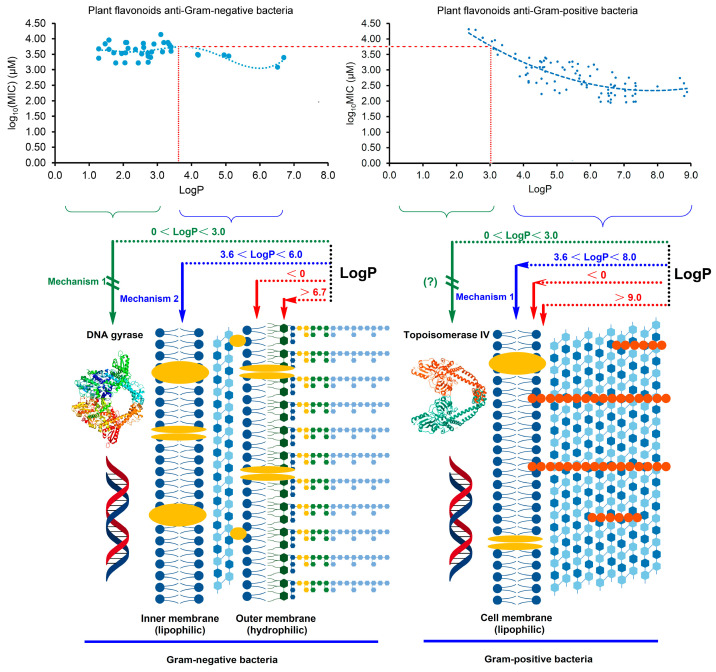
Schematic diagram of the interpretation for the different antibacterial effects and mechanisms of plant flavonoids against both Gram-negative and Gram-positive Bacteria, based on their characteristics of both regression curves shown in Figure 3 and their different cell structures.

Generally, when the LogP value is approximately between 0 and 3.0, the compound presents good cell membrane permeability to the lipid bilayer. As is well known, the cell envelope of Gram-negative bacteria contains a unique hydrophilic outer membrane and a lipophilic inner membrane which is similar to the cell membrane of Gram-positive bacteria. However, the cell structure of Gram-positive bacteria only contains a cell membrane, without an outer membrane. As shown in Figure 4, when the LogP was less than zero, it was difficult for plant flavonoids to approach the inner membrane of Gram-negative bacteria and the cell membrane of Gram-positive species to reach the target site. This indicates that plant flavonoids exhibit little antibacterial activity against both Gram-negative and Gram-positive bacteria. When the LogP value was between 0 and 3.0, it was easy for the plant flavonoids to cross the inner membrane of Gram-negative bacteria and the cell membrane of Gram-positive species. Thus, plant flavonoids can inhibit the DNA gyrase of Gram-negative bacteria, exhibiting a certain antibacterial activity, while their antibacterial effects on Gram-positive bacteria mainly relies on the membrane action, showing less antibacterial activity. When the LogP value was more than 3.6, it was difficult for the plant flavonoids to cross the inner membrane of Gram-negative bacteria, and their effect gradually changed from the inhibition of DNA gyrase to cell membrane action, including damaging the membrane and inhibiting respiratory chain, thus having similar effects on Gram-positive species. Therefore, their antibacterial activities against both Gram-negative and Gram-positive bacteria show similar trends with the increase in the LogP values between 3.0 and 6.0. However, the antibacterial activity of plant flavonoids against Gram-positive bacteria with the increase in the LogP value increased more rapidly than that against Gram-negative species. This is likely due to the optional electron acceptors such as ubiquinones and MKs in the quinone pool of Gram-negative bacteria, while there are only MKs in that of Gram-positive species. When the logP value was more than 6.7, it was difficult for plant flavonoids to access the inner membrane (action site) due to the obstruction from the hydrophilic outer membrane, and their antibacterial activities against Gram-negative bacteria gradually decreased. Similarly, their antibacterial activities against Gram-positive species also decreased, when the logP value was more than 9.0, even though there is no outer membrane in Gram-positive bacteria. This is likely due to the difficulty that plant flavonoids have in approaching the polarity head of the cell membrane when the logP value is too high. The above statement provides a reasonable interpretation for the characteristics of both regression curves in Figure 3, which, in turn, confirms the antibacterial mechanisms in this study and our previous report [20,21] of plant flavonoids against both Gram-negative and Gram-positive bacteria. Therefore, both the inhibition of DNA gyrase and action on the cell membrane, including membrane damage and the inhibition of the quinone pool of the respiratory chain, are two important mechanisms of plant flavonoids against Gram-negative bacteria. Additionally, their different antibacterial effects on Gram-negative and Gram-positive bacteria were attributed to these different antibacterial mechanisms and their cell envelope structure. 

Although both regression curves (Figure 2a,b) presented similar characters overall with the change in the LogP values, the MIC values of the tested plant flavonoids were obviously higher than those of the reported plant flavonoids. This was probably due to the different determination conditions, methods, details, and selected pathogenic strains. Among the tested and reported plant flavonoids, nine of them were the same. Thus, their antibacterial activities against *E. coli* ATCC 25922 were reorganized, and they are listed in Table S1, allowing for a comparison of the differences in the tested and reported data [25,28,29,32,34,38]. The results indicate that only the MICs of compound **13** (Licoflavone C) presented more than a 10 times difference between the tested (3026.36 μM) and reported (23.08 μM) values [29]. Therefore, using the broth microdilution method in 96-well plates, the antibacterial activity of this compound against *E. coli* ATCC 25922 was determined in six replicates, and all the MICs of compound **13** against *E. coli* ATCC 25922 were 3026.36 μM. 

As mentioned in Section 2.3, the antibacterial activities of plant flavonoids with logP values less than approximately 3.6 against Gram-negative bacteria were statistically stronger than those against Gram-positive species, and they also increased overall when the LogP value increased to more than approximately 3.6. However, the antibacterial activities of most plant flavonoids against Gram-negative bacteria were weak overall. Additionally, stronger antibacterial activities can be acquired through structural modification based on the antibacterial mechanism of plant flavonoids against Gram-negative bacteria, such as the inhibition of DNA gyrase and the quinone pool of the respiratory chain. However, due to the unique cell envelope structure of Gram-negative bacteria, it is challenging to discover plant flavonoid derivatives with very strong antibacterial activities against Gram-negative species. This is different from the case of Gram-positive bacteria, which does not have a hydrophilic outer membrane and only has the sole electron transfer acceptor menaquinone in the quinone pool [22]. Furthermore, when attempting to discover flavonoids with remarkably inhibitory activity against Gram-negative bacteria, it should also be encouraged to pay an attention to the combinational use of plant flavonoids and clinical antibiotics for combating AMR [7,31,34], as they have a good safety profile and can enhance susceptibility to many antibiotics.

## 4. Materials and Methods

### 4.1. Materials, Chemicals and Reagents

Thirty-seven plant flavonoids: icaritin (>98%), isoliquiritigenin (98%), formononetin (98%), isoliquiritigenin (98%), galangin (98%), baicalein (98%), diosmin (95%), hesperetin (97%), puerarin (98%), apigenin (≥95%), diosmetin (98%) and naringenin (97%) were purchased from Shanghai Macklin Biochemical Co., Ltd. (Shanghai, China); α-mangostin (>98.0%), licochalcone A (>98.0%), nobiletin (≥98.5%), tangeritin (≥98.5%), quercitrin (98%), sinensetin (98%), narirutin (98%), orientin (99%) and isoorientin (98%) were purchased from Chengdu Push Bio-technology Co., Ltd. (Chengdu, China); naringin (95%), neohesperidin (≥98%) and hesperidin (95%) were purchased from Shanghai Yuanye Bio-Technology Co., Ltd. (Shanghai, China); eriodictyol (≥98%), eriocitrin (≥98%), rhoifolin (≥98%) and licoflavone C (≥98%) were purchased from Wuhan ChemFaces Biochemical Co., Ltd. (Wuhan, China); glabridin (99.8%) and sophoraflavanone G (>98%) were purchased from Shanghai TopScience Co., Ltd. (Shanghai, China); quercetin (97%) was purchased from Meryer (Shanghai) Biochemical Technology Co., Ltd. (Shanghai, China); methyl-hesperidin (95%) was purchased from Shanghai Acmec Biochemical Co.,Ltd. (Shanghai, China); and didymin (≥98%), 5-demethylnobiletin (≥98%), 4’,5,7-trimethoxyflavone (≥98%), vitexin (≥98%) and isovitexin (≥98%) were purchased from Sichuan Weikeqi Biological Technology Co., Ltd. (Sichuan, China). All the compounds were stored at −20 °C. The stock solutions of the above plant flavonoids were prepared by dissolving them in a certain volume of dimethyl sulfoxide (DMSO) and diluting them with Mueller Hinton broth (MHB) to obtain a concentration of 4096, 8192, or 16,384 μg/mL. The stock solution was mixed well and then diluted to the desired concentration with MHB immediately before use. The concentrations of DMSO in all test systems were kept below 5.0%, and all those in the blank controls were 5.0%.

Casein hydrolysate (Qingdao Hope Bio-Technology Co., Ltd., Qingdao, China), starch soluble, beef extract, and agar powder (Sangon Biotech (Shanghai) Co., Ltd., Shanghai, China) were used to prepare the media. Mueller Hinton Agar (MHA) consisted of casein hydrolysate 17.5 g/L, starch soluble 1.5 g/L, beef extract 3.0 g/L, and agar powder 17.0 g/L, which were dissolved in purified water, and its pH value was maintained at 7.40 ± 0.20. MHB was prepared without agar powder according to the same composition and procedure as MHA. DMSO and 3-(4,5-dimethylthiazol-2-yl)-2,5-diphenyltetrazolium bromide (MTT) were purchased from Sangon Biotech (Shanghai) Co., Ltd. (Shanghai, China), and 96-well plates were purchased from Shanghai Excell Biological Technology Co., Ltd. (Shanghai, China). All reagents were of analytical or biochemical grade. All TopPette Pipettors (2~20 μL and 20~200 μL) were purchased from DLAB Scientific Co., Ltd., Beijing, China.

### 4.2. Bacterial Strains and Growth Conditions

*E. coli* ATCC 25922 and *S. aureus* ATCC 25923 were purchased from American Type Culture Collection, Manassas, VA, USA, and they were stored in Microbank^TM^ microbial storage (PRO-LAB diagnostics, Toronto, Canada) at −20 °C. Prior to use, *E. coli* and *S. aureus* were cultured onto an MHA plate at 37 °C, and then pure colonies from the culture plate were inoculated into MHB at 37 °C for 24 h on a rotary shaker (160 rpm) of a CHA-2 gas bath constant-temperature oscillator (Jintan Yineng Experimental Instrument Factory, Changzhou, China). A 1:100 dilution of the overnight culture was made into fresh MHB, and then incubated at 37 °C until the exponential phase for the following experiments. MHB was used for antimicrobial susceptibility tests. 

### 4.3. Antimicrobial Susceptibility Assay and Prediction

According to the standard procedure recommended by the Clinical and Laboratory Standards Institute (CLSI) [43], the exponential phase culture was diluted with MHB to achieve a bacterial concentration of approximately 1.0 × 10^6^ CFU/mL, and then the susceptibility of the plant flavonoids against *E. coli* ATCC 25922 or *S. aureus* ATCC 25923 was determined using the broth microdilution method on the 96-well plates in triplicate [7]. Based on the preliminary MIC values of the plant flavonoids, the initial concentration of each compound was set as 1024, 2048, or 4096 μg/mL. After the 96-well plate was incubated at 35 °C for 24 h, 20 μL of MTT (4.0 mg/mL) was added to each well, mixed well, and left for 30 min at ambient temperature. The MIC, defined as the lowest concentration of compounds that completely inhibited bacterial growth in the microwells, was assessed by observing no color change in the wells, while the blank wells had sufficient bacterial growth [22].

According to the reported antimicrobial quantitative relationship [20], the MICs of the plant flavonoids against Gram-positive bacteria *S. aureus* were also calculated using the equation *y* = −0.1285 *x*^6^ + 0.7944 *x*^5^ + 51.785 *x*^4^ − 947.64 *x*^3^ + 6638.7 *x*^2^ − 21,273 *x* + 26,087. Here, *y* is the antimicrobial activity (MIC) of the pant flavonoids against *S. aureus*; and *x* is the LogP value of the corresponding pant flavonoids, calculated using the software ACD/Labs 6.0 (Advanced Chemistry Development, Inc., Toronto, ON, Canada). Generally, the LogP values of the equation range from approximately 2.3 to 9.0. However, an appropriate extension of this range is also workable. It was considered acceptable for the predicted MIC value to fall into the range of 1/4 to 4 of the tested MIC value. Leniently, those MIC values ranging from 1/8× to 8× of the tested MIC value were also considered acceptable events, especially when the LogP value was outside the predicted range while close to both boundary values. This equation was established from 92 data pairs consisting of the LogP values and the MICs of plant flavonoids against Gram-positive bacteria, with an *R*^2^ value of 0.9413.

### 4.4. Structures and MICs of Reported Plant Flavonoids

As the antimicrobial activities of a certain compound against different pathogenic bacteria and strains vary, data on the chemical structures of plant flavonoids and their MIC against *E. coli* (a representative Gram-negative bacterium) were collected from the literature. Simultaneously, the tested strains of *E. coli* were sourced from American Type Culture Collection (ATCC) where possible. The structures, the MIC values against *E. coli*, and other related information of the plant flavonoids were unsystematically searched for on Google Scholar, and in several databases, such as SciFinder, Medline, Elsevier, ACS, ScienceDirect, Wiley Online Library, and Springer-Link, using the keywords “flavonoid” and “*E. coli*”, or and “antimicrobial”, or and “antibacterial”, and or and “Gram-negative bacteria”. Furthermore, the relevant references in the obtained literature were also tracked, and then the structures, the MIC values against *E. coli*, and other related information regarding the plant flavonoids were collected from the acquired literature. Finally, the structures of the collected compounds were drawn using the software ChemBioDraw Ultra 14.0.

### 4.5. Correlation and Regression Analyses

The lipophilic physicochemical parameters LogP (Log_10_ of partition coefficient) and LogD_7.40_ (Log_10_ of distribution coefficient at pH 7.40) of the plant flavonoids measured in Section 4.3 or previously reported were automatically calculated from their chemical structures using the software ACD/Labs 6.0 (Advanced Chemistry Development, Inc., Ontario, Canada). Subsequently, the MIC values (μM) and the LogP and LogD_7.40_ values of the plant flavonoids measured or reported were listed in an Excel table. After this, correlation analyses between the calculated LogP or LogD_7.40_ value (*x*) and the MIC (*y*) of the plant flavonoids in the table were performed using the software Microsoft Excel 2010 (Microsoft Corporation, Redmond, WA, USA). Simultaneously, corresponding regression equations were established for further verification, and the value of the correlation coefficient (*r*) was automatically calculated. It is noteworthy that the compounds (Table 2) without accurate MIC values (such as more than the measuring concentration) were not considered for the regression analyses, but their related information was used for the following discussion. Finally, the correlation between the antimicrobial activity MIC of the plant flavonoids against Gram-negative bacteria and their physicochemical parameter LogP or LogD_7.40_ was validated using the *r*-test. The coefficient of determination (*R*^2^) was also calculated to determine the fitting precisions according to Equation (5) in previous work [20].

### 4.6. Comparison of the Characteristics of Regression Curves

As referred to from our previous work [20], the MIC was further transformed to the log_10_(MIC), and then a regression analysis between the log_10_(MIC) (*y*) and the LogP or LogD_7.40_ (*x*) was performed. Then, the characteristics of the regression curves were compared on coordinate maps with the same vertical axis to explore the difference in the antibacterial effects and mechanisms of plant flavonoids against Gram-negative and Gram-positive bacteria, and to determine the possible reasons for the antibacterial effects and mechanisms changing with the lipophilic parameter LogP or LogD_7.40_ value. 

## 5. Conclusions

In summary, the antibacterial activities of plant flavonoids against Gram-negative bacteria are related to their lipophilicities, while their MIC values cannot be effectively predicted from their LogP values like in the case of Gram-positive bacteria. Simultaneously, the antibacterial activities of plant flavonoids against Gram-negative bacteria are generally weak, although most are stronger than those to Gram-positive species when the lipophilic parameter LogP value is less than approximately 3.0. Also, they are weak even when the LogP value is more than approximately 3.6. Unlike mainly acting on the cell membrane of Gram-positive bacteria, plant flavonoids probably have multiple action mechanisms against Gram-negative species, and among them the cell membrane is also an important action site. Moreover, DNA gyrase should also be considered another important target of plant flavonoids against Gram-negative bacteria.

## Figures and Tables

**Table 1:** Tested plant flavonoids together with their structure types, physicochemical parameters and antibacterial activities.

Compounds (No.) ^a^	Structure Types	LogP ^b^	LogD_7.40_^b^	*E. coli* ATCC 25922		*S. aureus* ATCC 25923	
				MIC (μM) ^c^	Log_10_(MIC)	MIC (μM) ^c^	Predicted MIC (μM) ^d^
Sophoraflavanone G (**1**)	Dihydroflavones	6.52	6.33	1206.15	3.0814	9.42	16.37
Neohesperidin (**2**)	Dihydroflavones	2.44	1.99	1677.12	3.2246	>1677.12	1816.01
Naringin (**3**)	Dihydroflavones	2.73	2.30	1763.88	3.2465	>1763.88	1151.90
Hesperidin (**4**)	Dihydroflavones	1.78	1.33	1677.12	3.2246	>1677.12	4440.63
Methyl hesperidin (**5**)	Dihydroflavones	2.54	2.10	3278.95	3.5157	>3278.95	8410.66
Eriodictyol (**6**)	Dihydroflavones	2.59	2.34	7104.70	3.8515	>7104.70	1442.76
Eriocitrin (**7**)	Dihydroflavones	1.47	1.03	>3433.13	>3.5357	>3433.13	6397.02
Hesperitin (**8**)	Dihydroflavones	2.90	2.65	6775.18	3.8309	>6775.18	863.94
Naringenin (**9**)	Dihydroflavones	3.19	2.96	7527.47	3.8766	3763.74	509.64
didymin (**10**)	Dihydroflavones	2.72	2.29	3444.51	3.5371	>3444.51	1170.98
Narirutin (**11**)	Dihydroflavones	2.07	1.65	3527.75	3.5475	>3527.75	3063.61
Baicalein (**12**)	Flavones	3.31	2.60	>3789.22	>3.5785	>3789.22	404.47
Licoflavone C (**13**)	Flavones	4.20	3.77	3026.36	3.4809	3026.36	85.1
Tangeritin (**14**)	Flavones	2.73	2.73	2749.95	3.4393	>2749.95	1151.9
Nobiletin (**15**)	Flavones	2.8	2.80	2544.73	3.4056	>2544.74	1025.21
Vitexin (**16**)	Flavones	1.28	0.45	2368.29	3.3744	2368.29	7888.23
Isovitexin (**17**)	Flavones	1.28	0.15	>2368.29	>3.3744	2368.29	7888.23
Diosmin (**18**)	Flavones	2.05	1.23	1682.69	3.2260	>1682.69	3146.25
Rhoifolin (**19**)	Flavones	1.72	0.91	3540.07	3.5490	>3540.07	4777.21
Apigenin (**20**)	Flavones	2.10	1.57	7578.45	3.8796	>7578.45	2942.82
Diosmetin (**21**)	Flavones	3.10	2.55	>6820.53	>3.8338	>6820.53	603.30
5-Demethylnobiletin (**22**)	Flavones	2.60	2.28	5273.32	3.7221	>5273.32	1420.23
4’,5,7-Trimethoxyflavone (**23**)	Flavones	3.35	3.35	6557.38	3.8167	>6557.38	373.96
Sinensetin (**24**)	Flavones	3.40	3.40	5499.91	3.7404	>5499.91	338.78
Orientin (**25**)	Flavones	1.58	0.72	>4567.55	>3.6597	>4567.55	5639.27
Isoorientin (**26**)	Flavones	1.58	0.41	4567.55	3.6597	>4567.55	5639.27
Quercetin (**27**)	Flavonols	2.07	1.40	>3388.04	>3.5299	13552.14	3063.61
Galangin (**28**)	Flavonols	2.83	2.16	3789.22	3.5785	>3789.22	974.47
Icaritin (**29**)	Flavonols	5.09	4.54	2779.66	3.4430	2779.66	75.22
Rutin (**30**)	Flavonols	1.95	1.22	>1677.26	>3.2246	1677.26	3585.77
Quercitrin (**31**)	Flavonols	2.36	1.63	4567.55	3.6597	>4567.55	2043.95
Isoliquiritigenin (**32**)	Chalcones	3.40	3.26	3995.94	3.6016	3995.94	338.78
Licochalcone A (**33**)	Chalcones	4.95	4.85	3026.00	3.4809	11.82	74.44
Formononetin (**34**)	Isoflavones	3.15	2.91	3817.05	3.5817	>3817.04	549.61
Puerarin (**35**)	Isoflavones	2.14	1.59	4918.58	3.6918	614.82	2787.56
Glabridin (**36**)	Isoflavanes	4.18	4.18	3156.79	3.4992	49.32	86.81
*α*-Mangostin (**37**)	Xanthones	6.70	6.10	2494.70	3.3970	4.87	8.17

^a^: The structures of these compounds are shown in Figure S1 in Supplementary Materials. ^b^: The LogP and LogD_7.40_ values were calculated using the software ACD/Labs 6.0. ^c^: MIC, minimum inhibitory concentration. ^d^: The MIC values of plant flavonoids were predicted from the equation y = −0.1285 x^6^ + 0.7944 x^5^ + 51.785 x^4^ − 947.64 x^3^ + 6638.7 x^2^ − 21,273 x + 26,087 [20], where y is the antimicrobial activity (MIC) of pant flavonoids against *S. aureus*; and x is the LogP value of corresponding pant flavonoids, also calculated using the software ACD/Labs 6.0.

**Table 2:** Reported plant flavonoids together with their structure types, physicochemical parameters, and inhibitory activities against *E. coli* ATCC 25922 [23,24,25,26,27,28,29,30,31,32,33,34,35,36,37,38].

Compounds (No.) ^a^	Structure Types	LogP ^b^	LogD_7.40_^b^	MIC (μM) ^c^	Log_10_(MIC) ^c^	References
Candidone (**38**)	Dihydroflavones	5.64	5.64	11.35	1.0540	[23]
Atalantoflavone (**39**)	Flavones	4.31	3.58	761.13	2.8815	[23]
2’-hydroxyatalantoflavone (**40**)	Flavones	3.87	3.10	726.57	2.8613	[23]
Neocyclomorusin (**41**)	Flavones	4.30	2.87	18.33	1.2632	[23]
Neobavaisoflavone (**42**)	Isoflavones	4.68	4.69	23.64	1.3736	[23]
Daidzein (**43**)	Isoflavones	3.14	2.61	236.83	2.3744	[23]
Isowighteone (**44**)	Isoflavones	4.78	4.23	380.57	2.5804	[23]
Isoneorautenol (**45**)	Dihydroisoflavones	4.16	4.16	794.14	2.8999	[23]
Abyssione-V 4′-*O*-methyl ether (**46**)	Dihydroflavones	8.03	7.79	9.23	0.9652	[24]
6,8-diprenylgenistein (**47**)	Isoflavones	7.33	7.16	19.19	1.2831	[24]
Alpinumisoflavone (**48**)	Isoflavones	5.80	4.93	11.60	1.0645	[24]
Eriodictyol (**49**)	Dihydroflavones	2.59	2.34	867.27	2.9382	[25]
Hesperetin (**50**)	Dihydroflavones	2.90	2.65	3308.19	3.5196	[25]
Neohesperidin (**51**)	Dihydroflavones	2.44	1.99	>1637.81	>3.2143	[25]
Neoeriocitrin (**52**)	Dihydroflavones	2.13	1.68	1341.07	3.1275	[25]
Naringin (**53**)	Dihydroflavones	2.73	2.30	>1722.53	>3.2362	[25]
Naringenin (**54**)	Dihydroflavones	3.19	2.96	2938.37	3.4681	[25]
5-hydroxy-7,4’-dimethoxyflavone (**55**)	Flavones	3.40	2.78	167.62	2.2243	[26]
Genkwanin (**56**)	Flavones	2.36	1.75	351.78	2.5463	[26]
Quercetin-5,3’-dimethylether (**57**)	Flavonols	2.30	1.74	151.38	2.1801	[26]
Rhamnazin (**58**)	Flavonols	2.51	1.73	302.76	2.4811	[26]
Rhamnocitrin (**59**)	Flavonols	2.56	1.82	166.52	2.2215	[26]
7,4’, 7’’, 4’’’-tetramethoxy amentoflavone (TMA) (**60**)	Bioflavones	5.80	4.10	420.47	2.6237	[27]
Isoginkgetin (IGG) (**61**)	Bioflavones	5.68	4.32	242.29	2.3843	[27]
Podocarpusflavone A (PFA) (**62**)	Bioflavones	4.15	2.53	452.5	2.6556	[27]
Nobiletin (**63**)	Flavones	2.80	2.80	439.86	2.6433	[28]
Kaempferol (**64**)	Flavonols	2.05	1.40	87.34	1.9412	[28]
Licoflavone C (**65**)	Flavones	4.20	3.77	23.08	1.3632	[29]
Derrone (**66**)	Isoflavones	5.55	4.82	23.22	1.3659	[29]
Epimedokoreanin B (**67**)	Flavones	6.59	6.14	>151.49	>2.1804	[30]
Auriculasin (**68**)	Isoflavones	7.70	6.89	9.51	0.9782	[30]
Pomiferin (**69**)	Isoflavones	7.44	7.06	19.03	1.2794	[30]
Gancaonin L (**70**)	Isoflavones	5.03	4.58	22.58	1.3537	[30]
Mopanin (**71**)	Flavonols	1.94	1.04	>214.59	>2.3316	[30]
Luteolin (**72**)	Flavones	2.40	1.85	349.36	2.5433	[31]
Quercetin (**73**)	Flavonols	2.07	1.40	1323.45	3.1217	[32]
Quercetin 3-*O*-*β*-D-glucosyl (1→4)-*α*-L-rhamnoside (**74**)	Flavonols	2.92	2.20	40.02	1.6023	[33]
Quercetin-3-*O*-*α*-L-rhamnoside or quercitrin (**75**)	Flavonols	2.51	1.78	108.13	2.0339	[33]
Entadanin (**76**)	Flavonols	2.00	0.086	36.31	1.5600	[33]
Kaempferide (**77**)	Flavonols	3.00	2.33	333.03	2.5225	[34]
Kaempferide-3-*O*-*β*-D-glucoside (**78**)	Flavonols	2.31	1.60	432.52	2.6360	[34]
Galangin (**79**)	Flavonols	2.83	2.16	370.04	2.5682	[34]
Tiliroside (**80**)	Flavonols	4.02	3.32	3.36	0.5263	[35]
Quercetin-3,7-*O*-*α*-L-dirhamnoside (**81**)	Flavonols	1.53	0.53	3.36	0.5263	[35]
Kaempferol-3,7-*O*-*α*-L-dirhamnoside (**82**)	Flavonols	1.73	0.76	3.46	0.5391	[35]
Scandenone (**83**)	Isoflavones	7.90	7.12	4.94	0.6937	[35]
Angusticornin B (**84**)	Chalcones	4.31	4.07	2.87	0.4579	[36]
Bartericin A (**85**)	Chalcones	5.46	5.77	1.49	0.1732	[36]
2’,4’,2-(OH)_3_-chalcone (**86**)	Chalcones	3.95	3.66	476.08	2.6777	[37]
2’,4’,3-(OH)_3_-chalcone (**87**)	Chalcones	3.35	3.05	179.51	2.2541	[37]
Isobavachalcone (**88**)	Chalcones	5.49	5.44	>394.6	>2.5962	[38]
*α*-Mangostin (**89**)	Xanthones	6.70	6.10	>311.84	>2.4939	[38]

^a^: The structures of these compounds are shown in Figure S1 in Supplementary Materials. ^b^: The LogP and LogD_7.40_ values were calculated using the software ACD/Labs 6.0. ^c^: MIC, minimum inhibitory concentration.

**Table 3:** Regression equations for the correlation between the physicochemical parameter (*x*) and the antimicrobial activity (*y*) against Gram-negative bacteria especially *E. coli*, of plant flavonoids.

Equation Number	Sample Numbers (*n*)	Parameters ^a^ (*x*)	Regression Equation (*r* ^b^)	Coefficient of Determination (*R*^2^)
(1)	37	LogP	*y* = 154.74*x*^4^ – 2328.1*x*^3^ + 11,774*x*^2^ – 23,630*x* + 20,574 (0.3736) ^c^	0.1396
(2)			*y* = −0.0022*x*^6^ + 0.0574*x*^5^ – 0.5624*x*^4^ + 2.6776*x*^3^–6.4916*x*^2^ + 7.5985*x* + 0.2396 (0.4714) ^d^	0.2222
(3)	37	LogD_7.40_	*y* = 51.533*x*^4^ – 648.46*x*^3^ + 2399.6*x*^2^ – 2834*x* + 5407.4 (0.3412) ^c^	0.1164
(4)	46	LogP	*y* = 3.4251*x*^6^ – 99.079*x*^5^ + 1128.5*x*^4^ – 6389*x*^3^ + 18,606*x*^2^ – 25,841*x* + 13,552 (0.4108) ^c^	0.1688
(5)			*y* = −0.0358*x*^4^ + 0.7264*x*^3^–5.2356*x*^2^ + 15.438*x*–13.244 (0.6670) ^d^	0.4449
(6)			*y* = 0.0621*x*^3^–0.9417*x*^2^ + 4.0824*x* – 2.9354 (0.5875) ^d^	0.3452
(7)	46	LogD_7.40_	*/* ^e^	*/*

^a^: The physicochemical parameter (*x*) was calculated using the software ACD/Labs 6.0. ^b^: *r*, correlation coefficient; the significant level *α* was set as 0.05, and the critical values of *r*_0.975_(35) and *r*_0.975_(44) were equal to 0.33 and 0.29, respectively. ^c^: The antimicrobial activity (*y*) was the MIC of a certain flavonoid against Gram-negative bacteria, especially *E. coli*. ^d^: The antimicrobial activity (*y*) was the log_10_(MIC) of a certain flavonoid against Gram-negative bacteria, especially *E. coli*. ^e^: No regression equation could be established since the *r* values are less than the critical one of *r*_0.975_(44).

## Data Availability

Data are contained within the article.

## Supplementary Materials

The following supporting information can be downloaded at: https://www.mdpi.com/article/10.3390/ph17030292/s1, Figure S1: Chemical structures of 52 plant flavonoids reported; Figure S2: Another effective regression curve for Equation (6) established from the LogP of the reported plant flavonoids and their log_10_(MIC) values; Figure S3: Polynomial regression analyses for the LogP (*x*) of plant flavonoids in Table S2 and the log_10_(IC_50_) (*y*) to DNA gyrase; Table S1: Comparison for the tested and reported MIC values of nine compounds [25,28,29,32,34,38]; **Table S2**: The ACD/LogP values and IC_50_ to DNA gyrase of 14 reported flavonoids, together with their MICs against *E*. *coli*_ss_ [39]; Table S3: Regression equations established from the LogP (*x*) values of the reported flavonoids [39] and the IC_50_ or Log_10_(IC_50_) (μM) values (*y*) of these flavonoids to DNA gyrase.
