# Supplementary material for: Antibacterial Activity and Mechanisms of Plant Flavonoids against Gram-Negative Bacteria Based on the Antibacterial Statistical Model
Source: Pharmaceuticals (Basel). 2024 Feb 24;17(3):292. doi: 10.3390/ph17030292 (PMC10974178; doi:10.3390/ph17030292)
Supplement: Supplementary file 1 [file pharmaceuticals-17-00292-s001.zip › pharmaceuticals-2853629-supplementary.pdf]

Supplementary Materials

Antibacterial Activity and Mechanisms of Plant Flavonoids against Gram-Negative Bacteria Based on the Antibacterial Statistical Model

Yu Yan [1,2,†], Xuexue Xia [1,2,†], Aiman Fatima [1], Li Zhang [2], Ganjun Yuan [1,2,*], Fengxian Lian [2] and Yu Wang [2]

[1] Biotechnological Engineering Center for Pharmaceutical Research and Development, Jiangxi Agricultural University, Nanchang 330045, China; yanyu99668@163.com (Y.Y.); xiaxuexue007@163.com (X.X.); aimanfatima222@hotmail.com (A.F.)

[2] Laboratory of Natural Medicine and Microbiological Drug, College of Bioscience and Bioengineering, Jiangxi Agricultural University, Nanchang 330045, China; zhangli43012022@163.com (L.Z.); 17848560041@163.com (F.L.); 15726263057@163.com (Y.W.)

* Correspondence: gyuan@jxau.edu.cn; Tel.: +86-0791-83813459

† These authors contributed equally to this work.

Figure S1. Chemical structures of 52 plant flavonoids reported.

Figure S2. Another effective regression curve for equation (6) established from the LogP of the reported plant flavonoids and their log_{10}(MIC) values.

Figure S3. Polynomial regression analyses for the LogP (x) of plant flavonoids in Table S2 and the $log_{10}(IC_{50})$ (y) to DNA gyrase.

Table S1. Comparison for the tested and reported MIC values of nine compounds.

Table S2. The LogP values and IC_{50} to DNA gyrase of 14 reported flavonoids, together with their MICs against *E. coli*$_{ss}$ [39].

Table S3. Regression equations established from the LogP (x) values of the reported flavonoids [39] and the IC_{50} or $Log_{10}(IC_{50})$ (μM) values (y) of these flavonoids to DNA gyrase.

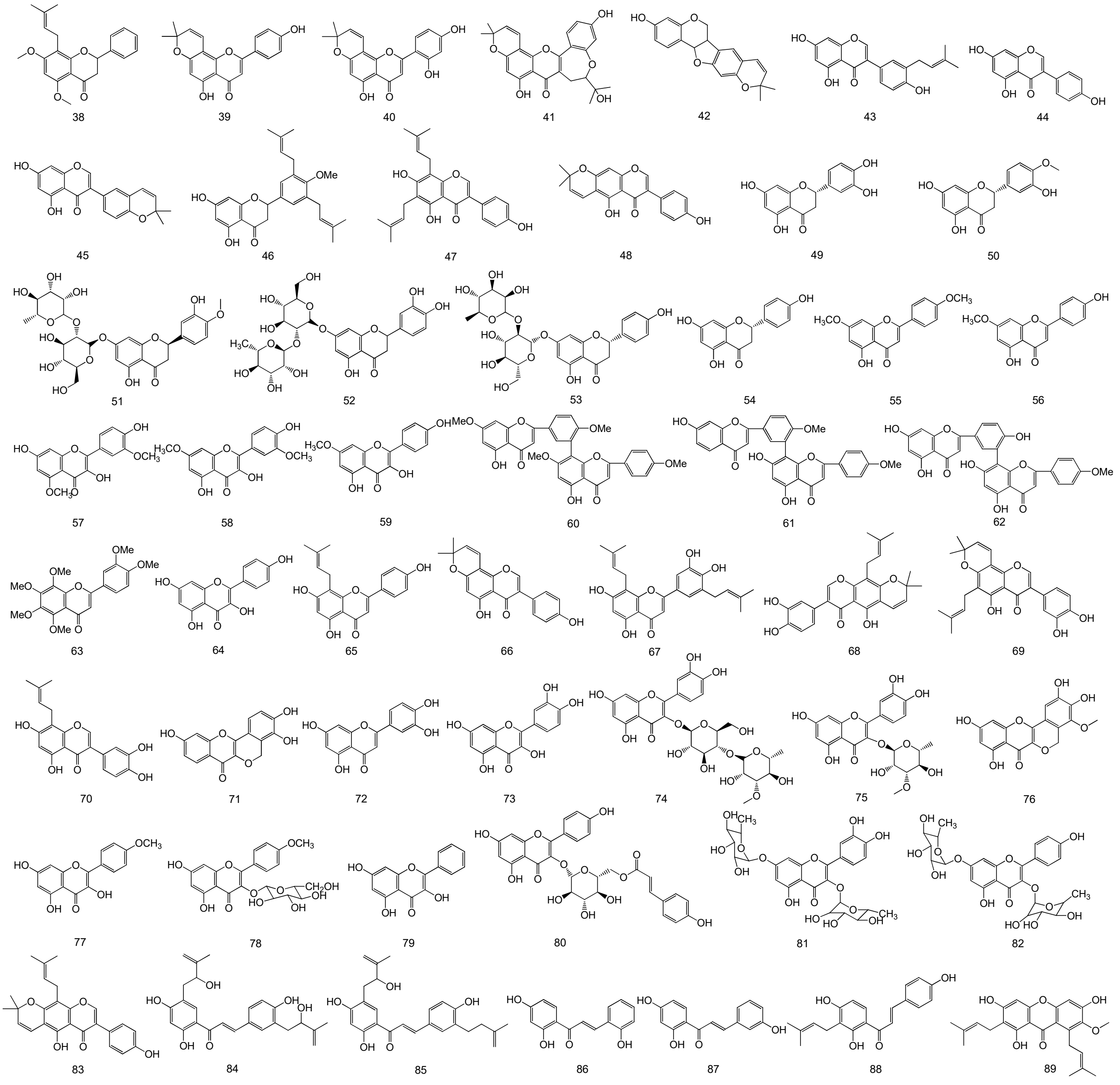

Figure S1. Chemical structures of 52 plant flavonoids reported.

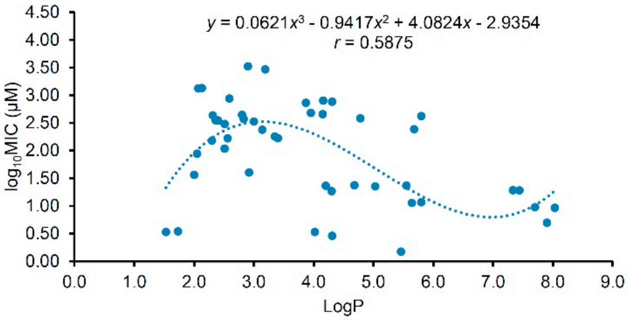

Figure S2. Another effective regression curve for equation (6) established from the LogP of the reported plant flavonoids and their $\log_{10}$(MIC) values.

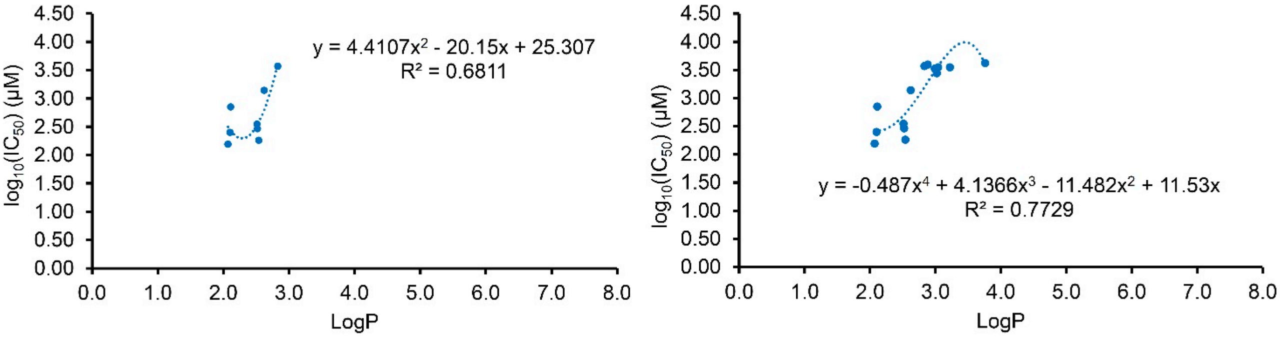

Figure S3. Polynomial regression analyses for the LogP (x) of plant flavonoids in Table S2 and the $\log_{10}(IC_{50})$ (y) to DNA gyrase. Left and right panels presented the regression curves respectively for equations (2) and (4) in Table S3.

Table S1. Comparison for the tested and reported MIC values of nine compounds.

Compound Number [a]	Compounds	Tested MIC (μM)	Reported MIC (μM)	References
**3** (**53**)	Naringin	1763.88	>1722.53	[25]
**6** (**49**)	Eriodictyol	7104.7	867.27	[25]
**8** (**50**)	Hesperetin	6775.18	3308.19	[25]
**9** (**54**)	Naringenin	7527.47	2938.37	[25]
**13** (**65**)	Licoflavone C	3026.36	23.08	[29]
**15** (**63**)	Nobiletin	2544.73	439.86	[28]
**27** (**73**)	Quercetin	>3388.04	1323.45	[32]
**28** (**79**)	Galangin	3789.22	370.04	[34]
**37** (**89**)	α-mangostin	2494.7	>311.84	[38]

[a]: The number out of bracket is the number of the tested flavonoids in Table 1, while that in bracket is the number of the reported flavonoids in Table 2.

Table S2. The LogP values and IC_{50} to DNA gyrase of 14 flavonoids reported, together with their MICs against *E. coli*$_{ss}$ [39].[a]

Compounds	LogP[b]	IC_{50} (μg/mL)	IC_{50} (μM)	$Log_{10}(IC_{50})$ (μM)[c]	MIC (μg/mL)
5a	2.07	47	155.51	2.192	31.25
5f	2.10	67.6	250.15	2.398	>125
5d	2.11	225	707.01	2.849	62.5
5n	2.51	89.2	350.85	2.545	125
5h	2.52	83.3	291.01	2.464	62.5
5k	2.54	55	181.97	2.260	62.5
5c	2.62	418	1383.01	3.141	125
5g	2.83	>500	>1850.21	>3.568	>125
5b	2.88	>500	>1966.65	>3.595	>125
5j	2.99	>500	>1665.17	>3.522	>125
5l	3.02	>500	>1387.66	>3.443	>125
5i	3.04	>500	>1758.89	>3.546	>125
5m	3.22	>500	>1758.89	>3.546	>125
5e	3.76	>500	>2098.72	>3.623	>125

[a]: The structures, IC_{50} (μg/mL), and MIC (μg/mL) of the designed flavonoids were reported by Ohemeng, *et al.*, and their MICs to *E. coli*$_{ss}$ were determined using the half dilution method [39].

[b]: The LogP values were calculated using the software ACD/Labs 6.0.

[c]: $log_{10}(IC_{50})$ means Log_{10} of IC_{50}.

Table S3. Regression equations established from the LogP (x) values of the reported flavonoids [39] and the IC_{50} or $Log_{10}(IC_{50})$ (μM) values (y) of these flavonoids to DNA gyrase.[a]

Equation number	Sample numbers (n)[b]	Regression equation (r[c])	Coefficient of determination (R^2)
(1)	8	$y = 15268x^3 - 99002x^2 + 212413x – 150519$ (0.9777)[d]	0.9559
(2)		$y = 4.4107x^2 - 20.15x + 25.307$ (0.8253)[e]	0.6811
(3)	14	$y = -4709.9x^3 + 40214x^2 - 108812x + 95023$ (0.9175)[d]	0.8418
(4)		$y = -0.487x^4 + 4.1366x^3 - 11.482x^2 + 11.53x$ [c] (0.8791)[e]	0.7729

[a]: The LogP (x) values, and IC_{50} and $Log_{10}(IC_{50})$ (μM) were shown in Table S2.

[b]: According to half dilution method, the IC_{50} of compound 5g was set as 1000 μg/mL and used for the regression analyses in equations (1) and (2), and while all compounds with the IC_{50} more than 500 μg/mL were set as 1000 μg/mL and used for those in equations (3) and (4), together with compounds 5a, 5f, 5d, 5n, 5h, 5k and 5c.

[c]: r, correlation coefficient; the significant level α was set as 0.05, and the critical values of $r_{0.975}$ (6) and $r_{0.975}$(12) were equal to 0.707 and 0.532, respectively.

[d]: Regression equations were established from the LogP (x) and the IC_{50} (μM) (y) values.

[e]: Regression equations were established from the LogP (x) and the $Log_{10}(IC_{50})$ (μM) (y) values.
